# Complementary and Alternative Medicine for Substance Use Disorders: A Scientometric Analysis and Visualization of Its Use Between 2001 and 2020

**DOI:** 10.3389/fpsyt.2021.722240

**Published:** 2021-11-05

**Authors:** Jiao Junyue, Chen Siyu, Wang Xindong, Xiao Qinge, Zeng Jingchun, Lu Liming, Lin Guohua

**Affiliations:** ^1^The First Clinical Medical College, Guangzhou University of Chinese Medicine, Guangzhou, China; ^2^Department of Rehabilitation, Liuzhou People's Hospital, Liuzhou, China; ^3^Department of Acupuncture, The First Affiliated Hospital of Guangzhou University of Chinese Medicine, Guangzhou, China; ^4^Clinical Medical College of Acupuncture Moxibustion and Rehabilitation, Guangzhou University of Chinese Medicine, Guangzhou, China

**Keywords:** acupuncture, bibliometrics [MeSH], complementary therapies, substance-related disorders, science map

## Abstract

**Background:** This study aimed to identify frontiers for further studies via brief understanding in complementary and alternative medicine (CAM) for substance use disorders (SUDs).

**Materials and Methods:** Publications on the use of CAM for treating SUDs were retrieved from the Web of Science Core Collection from 2001 to 2020 on July 12, 2020, and visualized by CiteSpace V.

**Results:** A total of 3,807 publications were obtained. The USA, China, and England were the leading research centers. However, India and Pakistan have recently focused on assessing CAM for the treatment of SUDs. Frederick L Altice was found to be the most productive author. *Addiction* ranked first among the frequently cited journals, which exceeded 1,000. The most common CAM therapies were acupuncture and CAM psychotherapies, such as mindfulness meditation.

**Conclusion:** CAM is gaining attention globally for treating SUDs. CAM psychotherapy and acupuncture are hotspots and deserve further study. Researchers should strengthen peer cooperation in this field.

## Background

Substance use disorders (SUDs) are a relapsing and remitting type of chronic encephalopathy associated with a cluster of cognitive, behavioral, and physiological symptoms, wherein the individual continues using legal or illegal substances, such as alcohol and nicotine, despite significant substance-related problems (1–3). Today, nearly 296 million people worldwide use drugs, a 28% increase from 2009 when over 35.6 million people experienced SUDs (4). Studies regarding the global burden of SUDs suggest that SUDs are the main contributors to the global burden of disability and mortality, especially those caused by tobacco and alcohol, followed by illicit drugs (5). In the USA, the government spends nearly $740 billion in medical care for SUDs annually (6, 7). SUDs contribute to incalculable international public health and societal loss; introduce challenges for the healthcare sector, social stability and safety, and community economy; and even cause personal, family, and financial problems.

A standard for the treatment of SUDs indicates that the treatment aims to stop or decrease drug use to improve health, recover normal social function, and reduce the risk of complications and relapse (8). It is acknowledged worldwide that SUDs are multifactorial diseases compounded with psychology, biology, psychopathy, pharmacology, and sociology, which need multidisciplinary, comprehensive, multi-sectoral collaborative treatment. Considering that the relapse rate of treatment is high despite recommended treatment guidelines, treatment services should be integrated and combined with complementary services. Complementary and alternative medicine (CAM) offers therapeutic practices that are not typically used in current conventional allopathic medical practice, such as diet, acupuncture, and meditation. The term “complementary” is defined as “used in addition to conventional treatments,” and “alternative” is defined as “instead of conventional treatment” (9, 10). Some evidence shows the effect of CAM practices, such as mindfulness meditation (MM) and motivational enhancement, in decreasing SUD relapse and substance-related injuries (11, 12). With some complementary and alternative therapies for SUDs, such as mind–body therapies, acupuncture, and meditation, proving to be effective, CAM is gaining momentum in addiction medicine (13, 14). However, some forms of CAM for SUDs still lack clear evidence regarding their efficacy to be recommended as treatment (15, 16). Further use of evidenced-based practices is required, such as acupuncture, meditation, and auricular needle; however, the number of people receiving SUD treatment is merely one in eight (4). Thus, an accurate picture of the research frontier and research trends is indispensable for studying the influence of CAM in the treatment of SUDs. In this study, we visualized a bibliometric analysis of related references by CiteSpace V to shed light on research trends and frontiers in the field of CAM for SUDs over the past 20 years. We present a brief understanding of research, development, and frontiers for further study.

## Materials and Methods

### Data Collection and Search Strategy

**Figure 1:**
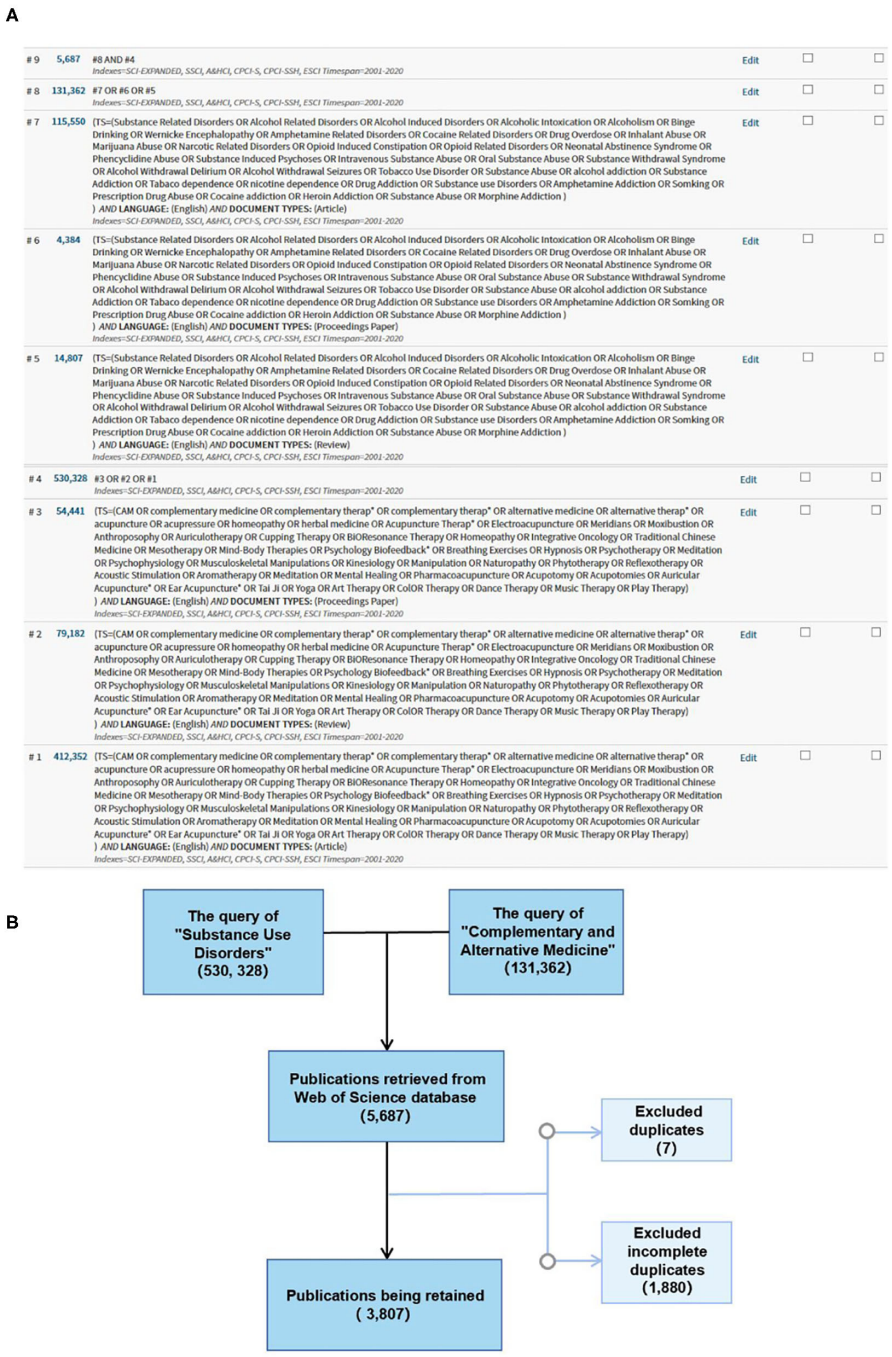
**(A)** Topic search queries and **(B)** flowchart of the inclusion of publications.

The collection of publications of CAM for SUDs involved two main steps. First, multiple topic search queries on the Web of Science made up the original data of this review. Several index terms related to “Substance Use Disorders” and “Complementary and Alternative Medicine” were listed from Medical Subject Headings (MeSH). All types of “SUD” and “CAM” were covered as much as possible. To ensure that current widely used methods for both topics were covered by our search query, using the “advanced search” option, the query was searched by text subject. The queries of “Substance Use Disorders” and “Complementary and Alternative Medicine” generated 530,328 and 131,362 records, respectively. Both were searched on July 12, 2020, in the Web of Science Core Collection (WoSCC) of studies from 2001 to 2020. Document types included the following: article, proceedings paper, review, and language-only English (Indexes=SCI-EXPANDED, SSCI, A&HCI, CPCI-S, CPCI-SSH, ESCI). We combined the two queries to find publications focused on the use of CAM for SUDs. A total of 5,687 records were obtained. The search queries are listed in Figure 1A.

### Analysis Tool

The study used CiteSpace V (5.6.R5), a Java-based software, to perform a bibliometric analysis for visualizing and analyzing the network (17–19). It can demonstrate the process of a systematic review based on a series of visual analytic functions implemented to help analyze knowledge inflection points, research hotspots, evolution paths, knowledge structures, and new trends in the knowledge field (18, 19). A set of main attributes of the knowledge base, including discipline composition, research tradition, keywords with content, and their interrelationships, together constitute the knowledge structure of the scientific field. The software supported several types of bibliometric studies, and co-occurrence analysis and visualization of the collaboration networks were performed (18, 20). As a result, the search for significant papers in the knowledge domain's literature was simplified (17).

### Methods and Analysis Process

The original data were downloaded as a plain text file. Those with full records and cited references comprised 5,687 records. The original data were filtered using a duplication function in CiteSpace, and seven duplicates and 1,880 incomplete publications were removed from the original data, resulting in 3,807 publications being retained, as shown in the flowchart in Figure 1B. The annual number of publications was calculated using the duplication function in CiteSpace software.

In the scientometric analysis, the cooperation, co-occurrence, and co-citation networks were analyzed by CiteSpace, including countries, institutions, authors, categories, keywords, cited journals, and references. For countries, institutions, and authors the top 200 in each year slice was calculated. For categories and keywords the top 100 and top 50 in each year slice, respectively, were set. For cited journals and references g-index was used to present the co-citation network on k = 25 and k = 30. The strongest citation burst was explored for countries, institutions, keywords, cited journals, and references by CiteSpace. The cluster analysis also used categories, keywords, and references by CiteSpace, which provided further knowledge regarding the hotspots in this field. All clusters were analyzed by keyword and log-likelihood ratio (LLR) tests. Keywords were visualized by the Timeline map to determine the change of keywords by time. Dual map, which is an overlay map showing the relationship in the field of published journals and cited journals, was analyzed by CiteSpace as well. To gain the most accurate results, synonyms for terms, such as “institutions,” “keywords,” and “cited journals,” were consolidated and manually added to an “Alias” list.

## Results

### Annual Publications

**Figure 2:**
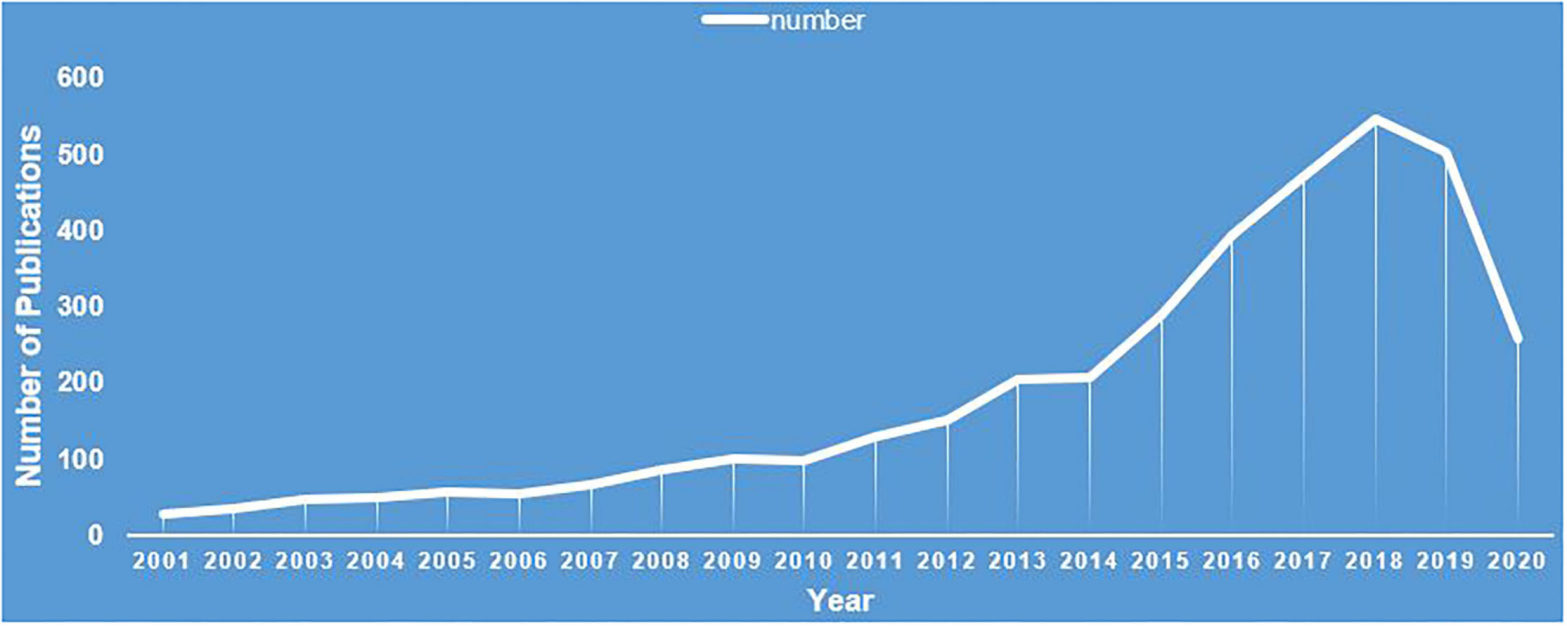
Annual number of publications on complementary and alternative medicine for substance use disorders.

In total, 3,807 records were included. The number of publications by year is presented in Figure 2, which shows that the number of publications has an overall upward trend, with a significant increase since 2015.

### Analysis of Countries

**Figure 3:**
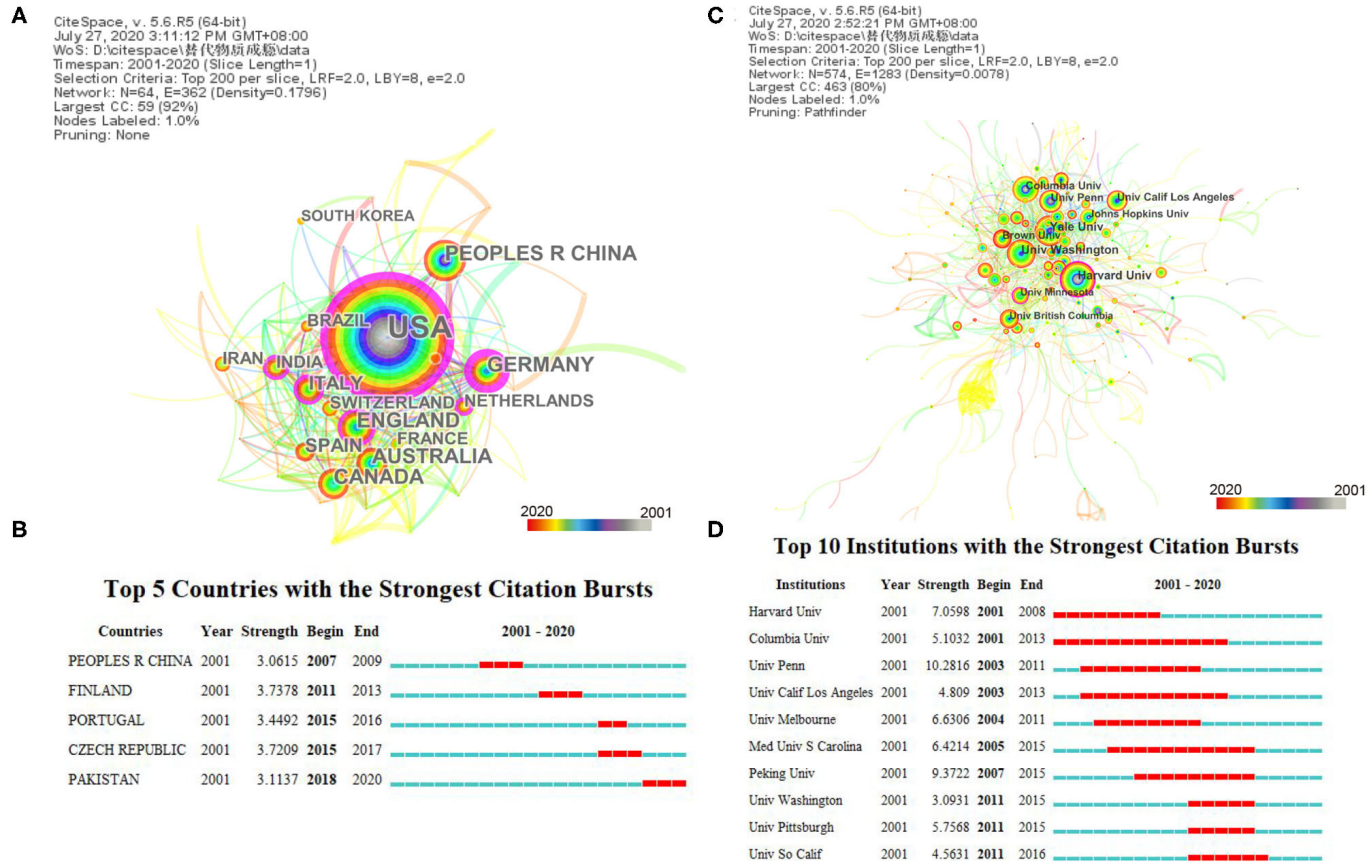
Visualization of complementary and alternative medicine (CAM) for substance use disorders (SUDs) by country and institution. **(A)** The network map of countries and regions for CAM for SUDs. A total of 64 countries or regions were involved, including 64 nodes and 362 links. **(B)** Top 5 countries with the strongest citation bursts. **(C)** The network map of CAM for SUDs by institutions. A total of 574 institutions participated in the publication, including 574 nodes and 1,283 links. **(D)** Top 10 institutions with the strongest citation bursts. **(A,C)** Top 15 results shown by time slice on 1 year, and different color presents different year as the legend shows. The nodes consist of an annual ring by different colors as legend, and the thickness of the annual ring represents the number of publications at the corresponding time. There is a purple ring in the outermost layers of some nodes, indicating that the node with high centrality may be a central node. **(B,D)** Each bar presents each year from 2001 to 2020. The red bar indicates that the institution had frequent citation bursts, and the blue bar indicates that the institution had infrequent citation bursts.

An analysis in terms of publication and centrality indicated that the USA, China, and England were the main research powers in the research into the use of CAM for SUDs. Among them, the USA had the highest sigma and centrality (Figure 3A and Table 1), nearly nine times that of China, which means the most influential country is involved in this field. As Figure 3A and Table 1 present, the major countries are developed countries in North America and Europe. However, developing countries, such as China, India, and Pakistan, also report significant studies. Burst detection in Figure 3B found five countries: China, Finland, Portugal, Czech Republic, and Pakistan. Moreover, the research from Pakistan has been bursting high since 2018.

**Table 1:** Top 15 countries/regions related to complementary and alternative medicine for substance use disorders.

**Rank**	**Freq**	**Centrality**	**Author**	**Half-life**
1	2,035	0.32	USA	15
2	230	0.09	Peoples R China	13
3	211	0.15	United Kingdom	15
4	207	0.02	Canada	12
5	186	0.2	Germany	15
6	156	0.07	Australia	14
7	136	0.15	Italy	14
8	107	0.04	Spain	13
9	84	0.11	Netherlands	8
10	77	0.18	India	9
11	76	0.03	France	8
12	72	0.03	Brazil	9
13	66	0.03	Switzerland	14
14	61	0.02	Iran	5
15	51	0	South Korea	7

### Analysis of Institutions

Of the top 15 institutes that paid close attention to the field of CAM for SUDs, 14 institutes were in the USA, including Yale University, Harvard University, and the University of Washington (Figure 3C and Table 2). In addition, only the University of British Columbia in Canada ranked 10th on the list. Thus, the frontier institutes were mainly found on North America. The institution with the highest strength and a strong citation burst was the University of Pennsylvania, followed by Peking University and Harvard University (Figure 3D). Harvard University had the highest centrality, which is a central node in the structure preference to cooperation across institutions. Thus, the University of Pennsylvania and Harvard University have bigger influence and field frontier topics.

**Table 2:** Top 15 institutions related to complementary and alternative medicine for substance use disorders.

**Rank**	**Freq**	**Centrality**	**Institution**	**Half-life**
1	107	0.04	Yale Univ	12
2	97	0.14	Harvard Univ	15
3	96	0.04	Univ Washington	10
4	74	0.06	Columbia Univ	15
5	65	0.06	Univ Penn	12
6	63	0.03	Univ Calif Los Angeles	13
7	60	0.06	Brown Univ	13
8	60	0.04	Johns Hopkins Univ	14
9	47	0.1	Univ Minnesota	7
10	46	0.02	Univ British Columbia	7
11	45	0.08	Univ Calif San Diego	6
12	43	0.03	Univ N Carolina	8
13	42	0.08	Boston Univ	7
14	39	0.02	Univ Michigan	5
15	37	0.01	Univ Miami	7

### Analysis of Authors

**Figure 4:**
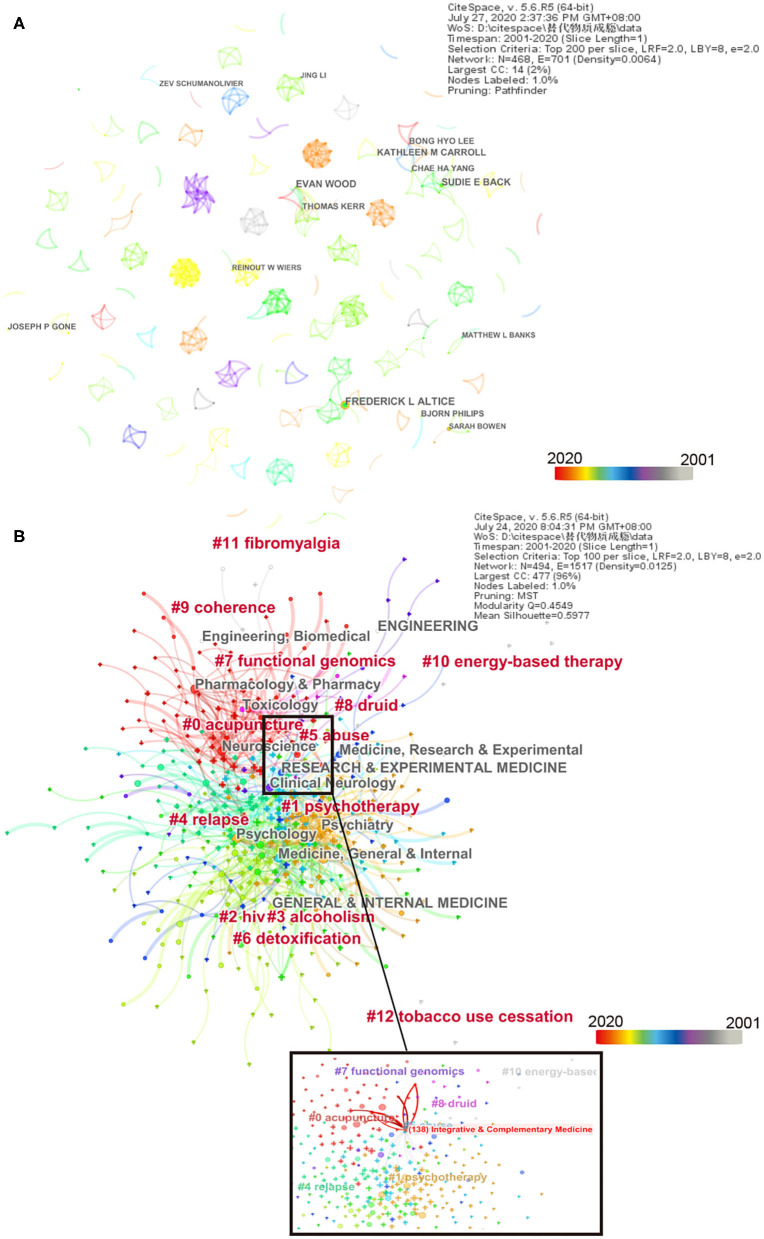
Network map of complementary and alternative medicine (CAM) for substance use disorders (SUDs) by author and category. **(A)** Regarding the network map by authors, top 14 results present CAM for SUDs. A total of 468 authors participated, including 468 nodes and 701 links. **(B)** The co-occurrence network map of the cluster by category (top 10 result); 494 nodes and 1,517 links were included. The modularity Q is 0.4549, and the mean silhouette is 0.5977. Time slice on 1 year, and different colors present different years as the legend shows. The color lines between authors indicate their first cooperation time, and the thickness of the lines indicates the times of authors' cooperation. The area in similar color suggests similar clusters and shows cluster at the corresponding time as well.

Table 3 and Figure 4A show that of the number of publications, Frederick L. Altice was the most productive author, followed by Evan Wood, Sudie E. Back, Kathleen M. Carroll, and Bong Hyo Lee. In terms of active time, Kathleen M. Carroll and Bong Hyo Lee both had the longest half-life of 6, having long active time and influence.

**Table 3:** Top 15 authors related to complementary and alternative medicine for substance use disorders.

**Rank**	**Freq**	**Author**	**Half-life**
1	14	Frederick L. Altice	2
2	11	Evan Wood	3
3	11	Sudie E. back	2
4	10	Kathleen M. Carroll	6
5	8	Bong Hyo Lee	6
6	8	Thomas Kerr	4
7	7	Chae Ha Yang	4
8	7	Bjorn Philips	3
9	7	Joseph P. Gone	1
10	6	Reinout W. Wiers	2
11	6	Sarah Bowen	2
12	6	Jing Li	3
13	6	Matthew L. Banks	0
14	6	Zev Schumanolivier	4
15	5	S. Stevens Negus	2

### Analysis of Categories

The research on the treatment of SUDs using CAM mainly involves multi-subject areas, including psychiatry, psychology, engineering, pharmacology and pharmacy, toxicology, neuroscience, research and experimental medicine, clinical neurology, and general and internal medicine. The top 10 clusters by keywords were acupuncture (#0), psychotherapy (#1), HIV (#2), alcoholism (#3), relapse (#4), abuse (#5), detoxification (#6), functional genomics (#7), druid (#8), and coherence (#9) (Figure 4B). Acupuncture and psychotherapy are nearly red, suggesting the two topics are trending or have hot treatment in this field in recent years.

### Analysis of Keywords

**Figure 5:**
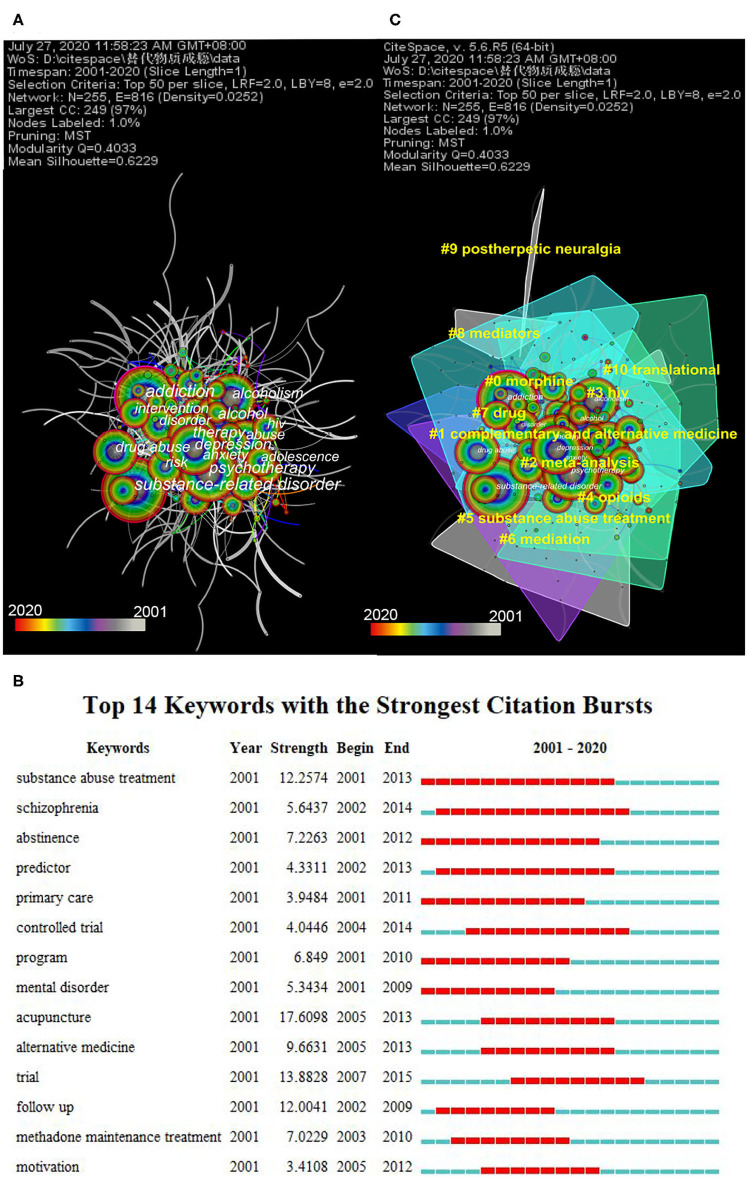
Visualization of complementary and alternative medicine (CAM) for substance use disorders (SUDs) by keyword. **(A)** The network map of keywords (top 15 results); 255 keywords were included, with 255 nodes and 816 links. **(B)** Top 14 keywords with the strongest citation bursts. Each bar presents each year from 2001 to 2020. The red bar indicates that the institution had frequent citation bursts, and the blue bar indicates that the institution had infrequent citation bursts. **(C)** The co-occurrence network map of the cluster by keywords for CAM for SUDs. A total of 16 clusters were found, with a modularity Q of 0.4033 and a mean silhouette of 0.6229. Time slice on 1 year, and different colors present different years as the legend shows. The nodes consist of an annual ring by different colors as the legend, and the thickness of the annual ring represents the frequency of keywords at the corresponding time. The area with similar color suggests similar clusters and shows cluster at the corresponding time as well.

The top 15 keywords from the top 50 on each year from 2001 to 2020 are shown in Figure 5A and Table 4. In terms of frequency and centrality, psychotherapy was a research hotspot, suggesting that although depression, anxiety, and HIV are treated with CAM for SUDs concerning comorbidities, CAM used among adolescents with SUDs is also a concern. In keyword citation burst detection analysis in Figure 5B, acupuncture (strength: 17.6098) had the greatest strength burst in this field from 2005 to 2013, followed by trial, substance abuse treatment, follow-up, alternative medicine, and abstinence. Complementary therapy for SUDs, especially acupuncture, is not widely used, regardless of its efficiency. Moreover, the involvement of adolescents in CAM for SUDs is considered distressing (21–23).

**Table 4:** Top 15 keywords related to complementary and alternative medicine for substance use disorders.

**Rank**	**Freq**	**Centrality**	**Keyword**
1	818	0.16	Substance-related disorder
2	711	0.23	Addiction
3	435	0.08	Psychotherapy
4	384	0.1	Depression
5	380	0.06	Therapy
6	355	0.08	Alcohol
7	309	0.07	Alcoholism
8	262	0.12	Drug abuse
9	225	0.02	Anxiety
10	214	0.05	Disorder
11	214	0.05	Risk
12	205	0.08	Abuse
13	198	0.06	HIV
14	195	0.12	Intervention
15	183	0.05	Adolescence

Cluster network analysis of keywords was performed using the LLR test (24). In the cluster network, Q values represent the modularization of the network. The larger the Q value, the better the clustering of the network, which is significant at Q > 0.3. Silhouette is an index to measure the homogeneity of a cluster. The larger the value, the higher the similarity. It indicates high reliability when the silhouette value is >0.7 (25). Size represents the number of members in the cluster (25). The mean year represents the average year of publications in the cluster. The key clusters of keywords are shown in Supplementary Table 1 and Figure 5C. Morphine (#0) was identified as the main substance in this field, with a long-term hot issue for the entire period from 2001 to 2020, and relapse, acupuncture, electroacupuncture (EA), and psychotherapy were most relevant to it. Research on CAM (#1) generally co-appeared with efficacy, outcome, placebo-controlled trials, and auricular acupuncture. HIV (#3) also received significant attention as an infectious disease highly related to drug abuse. The meta-analysis (#2), as the highest standard of evidence-based medicine, was more often with women, youth, culturally sensitive interventions, prevalence, depression, and psychotherapy. Opioids (#4) were closely associated with pharmacology, pain management, opioid, and pain and were the most frequently prescribed initial treatment for post-therapeutic neuralgia (#9). Meditation (#7) was used for a short time, with alcoholism treatment, psychoactive SUD, and randomized clinical trials.

### Analysis of Cited Journals and Dual Map

**Figure 6:**
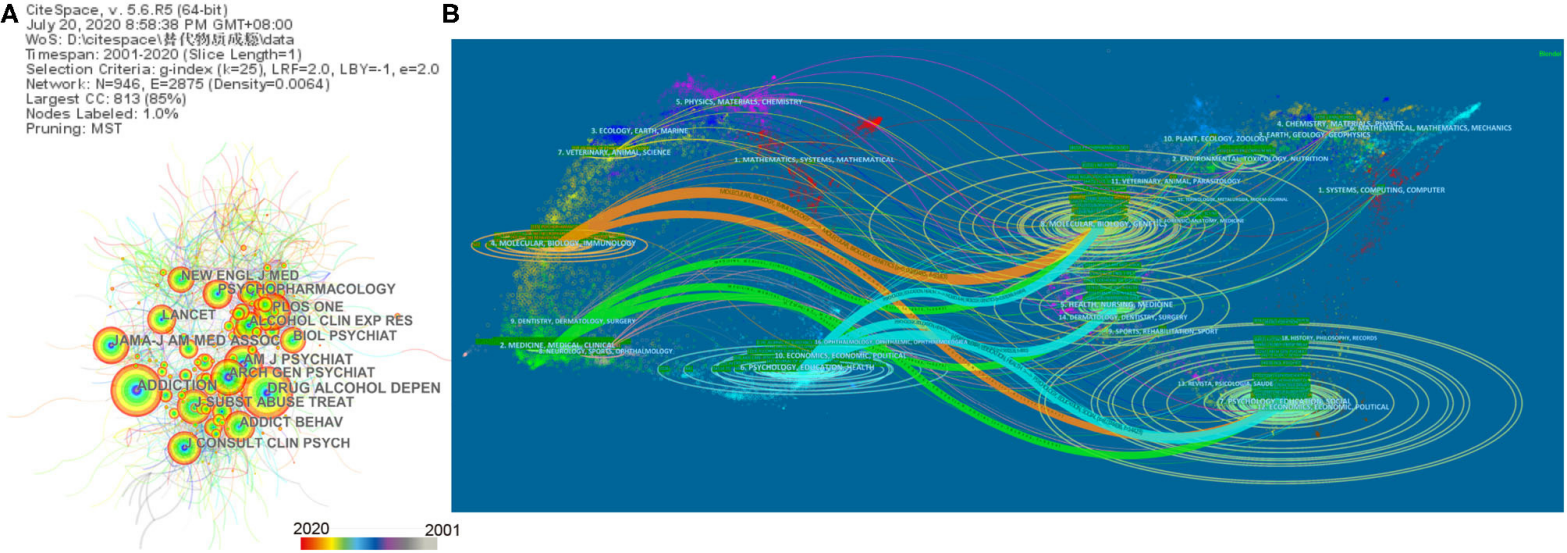
Network map and dual map of cited journals for complementary and alternative medicine (CAM) for substance use disorders (SUDs). **(A)** Network map and dual map of the cited journals for CAM for SUDs (top 15 results). A total of 944 journals were cited, with 946 nodes and 2,875 links. Time slice on 1 year, and different colors present different years as the legend shows. The nodes consist of an annual ring by different colors as the legend, and the thickness of the annual ring represents the number of research published on the journal at the corresponding time. **(B)** A dual map of journals of published papers. Lines indicate cross studies between disciplines, and the thickness of the lines indicates close connections. The orange path: Papers published in molecular/biology/immunology, mostly cited journals in molecular/biology/genetics and psychology/education/social. The green and blue paths: Papers published in medicine/medical/clinical journals and psychology/education/health journals mostly cited in molecular/biology/genetics, psychology/education/social, and health/nursing/medicine.

A cited journal map (Figure 6A) was generated for 944 journals. *Addiction* ranked first in frequency for cited journals, followed by *Drug and Alcohol Dependence, JAMA Psychiatry*, and *American Journal of Psychiatry*, which exceeded 1,000. It should be noted that the *Archives of General Psychiatry* changed its name to *JAMA Psychiatry*; thus, the two terms were combined. As Table 5 presents, the cited journals are mainly in the field of addiction medicine and psychiatry. Figure 6B shows that the majority of journals that published papers in this field include three subjects: molecular/biology/immunology, medicine/medical/clinical, and psychology/education/health, especially psychology/education/health. The majority of journals that cited papers in this field also included three subjects: molecular/biology/genetics, psychology/education/social, and health/nursing/medicine, especially psychology/education/social, which had the most authors and papers.

**Table 5:** Top 15 cited journals related to complementary and alternative medicine for substance use disorders.

**Rank**	**Cited times**	**Cited Journal**	**IF 2019**
1	1,304	Addiction	6.343
2	1,303	Drugs and Alcohol Dependence	3.951
3	1,068	American Journal of Psychiatry	14.119
4	993	JAMA-journal of the American Medical Association	45.54
5	951	Archives of General Psychiatry/ JAMA Psychiatry	17.471
6	933	Journal of Consulting and Clinical Psychology	4.632
7	913	PLoS ONE	2.74
8	903	Journal of Substance Abuse Treatment	3.083
9	872	Addictive Behaviors	3.645
10	841	Psychopharmacology	3.13
11	777	The Lancet	60.392
12	756	Alcoholism: Clinical and Experimental Research	3.035
13	739	The New England Journal of Medicine	74.699
14	724	Biological Psychiatry	12.095
15	674	Proceedings of the National Academy of Sciences	9.412

### Analysis of Reference

**Figure 7:**
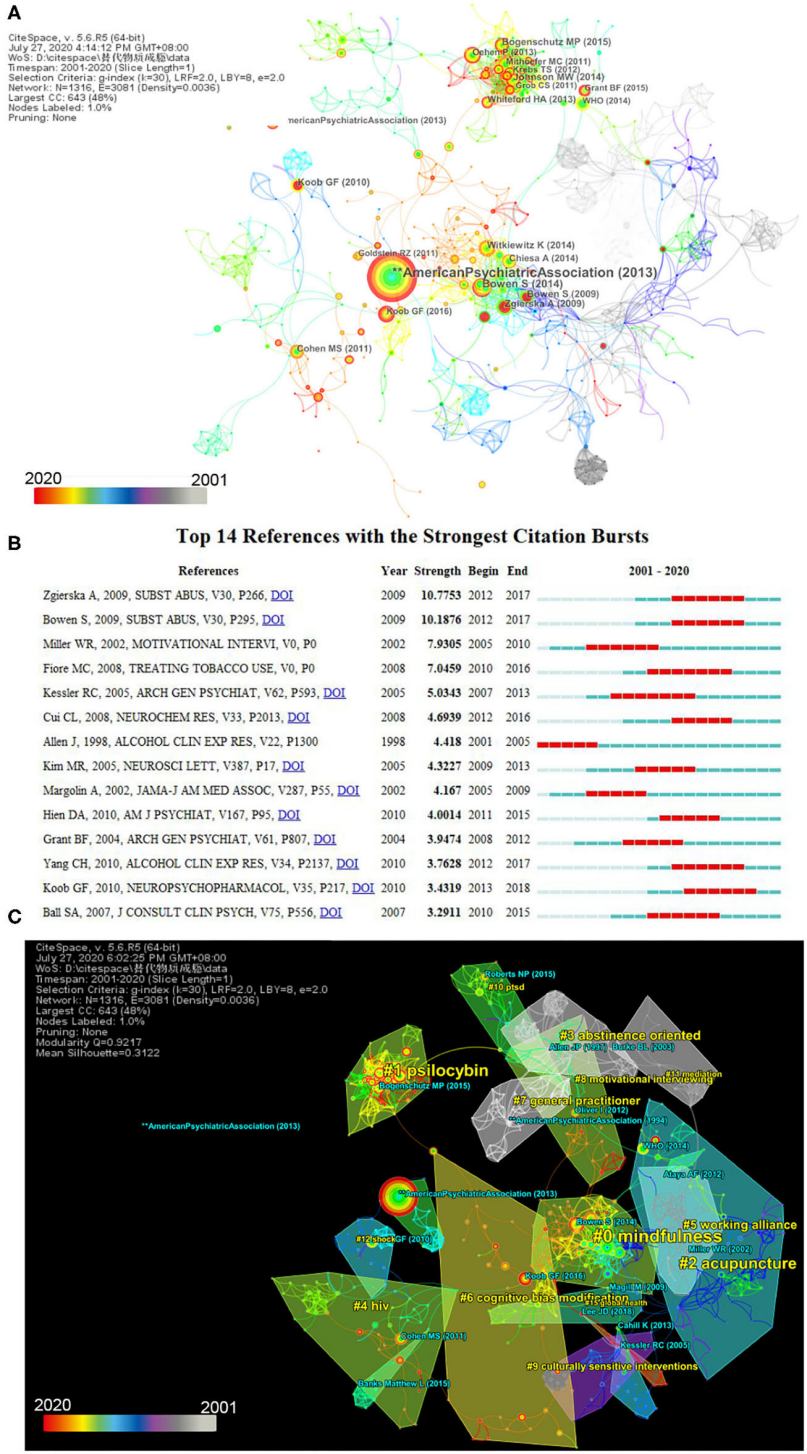
Visualization of references for complementary and alternative medicine (CAM) for substance use disorders (SUDs). **(A)** The network map of cited references for CAM for SUDs (top 15 results). A total of 1,316 references were cited, with 1,316 nodes and 3,081 links. **(B)** The co-occurrence network map of the cluster of references. The modularity Q was 0.9217, and the mean silhouette was 0.3122. **(C)** Top 14 references with the strongest citation bursts. Each bar presents each year from 2001 to 2020. The red bar indicates that the institution had frequent citation bursts, and the blue bar indicates that the institution had infrequent citation bursts. Time slice on 1 year, and different colors present different years as the legend shows. The nodes consist of an annual ring by different colors as the legend, and the thickness of the annual ring represents times of cited study at the corresponding time. The area with similar color suggests similar clusters and shows cluster at the corresponding time as well.

We ranked the top 10 cited references by frequency (Supplementary Table 2 and Figure 7A) or centrality (Supplementary Table 3). These are the most cited study or the most influential study during the period. As stated in the previous section (“Analysis of cited journals and dual map”), the American Psychiatric Association published the 5th Diagnostic and Statistical Manual of Mental Disorders in 2013, with its article having the highest frequency of citation, and the study by Gifford et al. in 2006 had the highest centrality. Combined with burst citation (Figure 7B), Bowen et al. had a strong burst, sigma, frequency, and half-life.

Although the mean silhouette of the network of the cluster in Figure 7C was 0.3122, which is < 0.5, the number of mean silhouettes of each cluster was >0.957 overall, which suggests that the cluster is rational and credible. The network shows that #0, #2, #6, #8, #9, and #11 are interventions for SUDs, including mindfulness, acupuncture, cognitive bias modification, motivational interviewing, and meditation, which are the major areas cited in research. However, mindfulness, cognitive bias modification, and motivational interviewing are psychotherapies, which may mean CAM crosses much with psychotherapy in this field. Mindfulness, the largest cluster (#0), with a size of 88, mostly focused on the relationship between mindfulness and relapse, followed by evidenced-based medicine, sustaining a high citation rate. Psilocybin (#1), with a size of 79 and the second-largest cluster, has been trending in the recent years, which is indicated by the increasing redness of the lines and circles the closer it gets to the current year, eventually turning completely red. Acupuncture (#2), with a size of 70, the third cluster, had four burst articles.

## Discussion

### General and Collaboration Information

Overall, the number of publications has been increasing annually, especially since 2015; although it decreased in 2019, it is gaining global attention. From the trend of the publications' numbers, we discovered that several studies were conducted on CAM for SUDs. CAM for SUDs is gaining attention worldwide.

An analysis of countries and institutions shows that the studies on CAM for SUDs are mainly conducted in developed countries (8 of the top 10), especially North America, which has a high centrality and publishing number. However, CAM for SUDs has also been a topic of focus in developing countries, such as China, India, and Pakistan, which provide increased attention to and collaboration on the topic and have comparatively greater centrality. Pakistan is still in the citation burst. The increasing concern of developing countries is to determine which illicit substances and drugs are associated with SUDs to reduce SUDs in those countries, as the United Nations Office on Drugs and Crime reported. Except for the University of British Columbia, the top leading research institutions are in the USA, such as Yale University, Harvard University, and the University of Washington. Although Harvard University is the second most common parent institute in a number of publications, it had the highest betweenness centrality (0.14), sigma (2.47), and the longest half-life (15), having the largest influence and a long-time history in this field, and preferred cross-institution cooperation. The author's analysis showed that the most productive author was not the most influential author. Kathleen M. Carroll and Bong Hyo Lee had a long-time influence. The collaboration is intimate among institutions. However, there is little collaboration among authors in this field, and most collaboration between authors is dominated by small groups lacking cross-team cooperation. Authors should increase collaboration across institutions and countries. The studies on the treatment of SUDs using CAM mainly involve multi-subject areas.

### Citation Information

The most published journals were in psychology/education/health, and the most frequently cited journals were in psychology/education/social. Moreover, papers published in molecular/biology/immunology and papers cited in molecular/biology/genetics cannot be ignored. Thus, the aforementioned disciplines intersected closely with each other, especially molecular/biology/immunology with molecular/biology/genetics and psychology/education/health with psychology/education/social. Among them, the *New England Journal of Medicine* had the highest impact factor (IF; 74.699), and among the top 10 cited journals, more than half of the journals had IF higher than 5, which suggests that research in this field is of high quality. From the results of the most cited journals, journals about CAM, such as *BMC Complementary Medicine and Therapies, The Journal of Alternative and Complementary Medicine*, and *The American Journal of Chinese Medicine*, were all present in the results data but not frequently sufficient to include in the top 15, which shows that researchers in this field prefer reference papers on addiction medicine. The article with the highest frequency was the American Psychiatric Association publishing the 5th Diagnostic and Statistical Manual of Mental Disorders in 2013, which means it is an acknowledged diagnostic standard in the field. The highest centrality cited study was written by Gifford et al. in 2006 about a model to calculate the influence of relationship networks on patients with SUD (26).

### Research Fields and Frontiers

From the category and dual map analysis, we could easily identify the main fields in CAM for SUDs: psychology, education, and health in journals, research, and experimental medicine. In Figure 4B, integrative and complementary medicine was in the center and multi-crossed with other disciplines, including partly acupuncture and psychotherapy. By category, cluster keywords indicate acupuncture and psychotherapy are hot topics in this field, and CAM is associated with psychotherapy. From the research directions of high-frequency authors, we found the research directions involved in CAM for SUDs. Frederick L. Altick focused on opioid substitution therapy, HIV comorbidity, and neuroscience mechanism (27, 28). Bong Hyo Lee and Chae Ha Yang collaborated for experimental animal research on acupuncture for SUDs (29, 30). Bong Hyo Lee, who had the longest half-time, focused on drug addiction and withdrawal, such as the peripheral mechanisms with acupuncture treatment for drug addiction (31, 32). Interestingly, the Peking University, a burst citation institution, was interested in traditional Chinese medicine for SUDs in clinical and laboratory settings, such as acupuncture, EA, and herbal medicines (33–35).

Through the results of keyword analysis and burst, research hotspots and frontiers in the field of CAM for SUDs were found. As the clusters and keyword frequency show, substance abuse involving morphine and opioids has always been a research attraction (36). HIV is the main comorbidity with substance abuse (37). SUD is more prevalent in people with HIV than in the general population, with regard to the disordered use of almost all substances, including alcohol, stimulants, opioids, and tobacco (38). Of all substances, heroin and cocaine are the drugs most closely related to HIV infection, and the use of amphetamines is increasing (39). Drug addiction is increasing among adolescents and young adults, which includes addiction to tobacco, alcohol, and illicit drug use, which is a risk factor (40). Because adolescents are in a vulnerable period for the development of substance abuse, it will result in SUD adulthood, and adolescents are at risk of being criminals because of drug addiction, which also draws great attention in this field. Depression and anxiety are comorbidities with substance abuse and are high-frequency keywords as well; moreover, they may increase the risk of repeated drug usage in large quantities. SUD may persist even if depression and anxiety are properly treated or relieved (41). As Figures 5, 7 present, CAM and substance abuse treatments are significant concerns, particularly psychotherapy and acupuncture. The psychotherapy of drug abuse mainly focuses on mind–body therapies, such as meditation, and various other types of behavioral therapy that have been proven to be effective interventions (42). However, psychotherapy is associated with not only CAM but also psychotherapies, especially complementary and alternative psychotherapies (CAPs), and MeSH terms related to some psychotherapies, such as “biofeedback, psychology,” or “imagery, psychotherapy,” may produce similar results (43). The word “psychotherapy” may change its definition in recent years. In those papers (44, 45), the list or definition of CAM partly contained psychotherapy; however, psychotherapy prefers to be separated from CAM in recent days. Moreover, it might indicate that multiple psychotherapies are interactive with CAM. Clusters of CAM, including some psychotherapy, were also observed, which may show how CAM is closely associated with psychotherapy for SUDs. Thus, psychotherapy in this result may mainly indicate CAP or CAM integration with psychotherapy. Although the efficacy of CAM with psychotherapy remains controversial, there are researchers suggesting that 65% of respondents indicated that they had used at least one form of CAM in a study year by themselves (46–48). Particularly in patients with SUDs, who have complicated pathogenic factors and symptoms, comprehensive treatment is better. CAM integrated with psychotherapy or CAP may be a hotspot in this field, like MM. Acupuncture is an oriental medicine that includes auricular acupuncture and EA. Evidence from experiments and clinics has confirmed that it has an improved effect on SUDs (49, 50). Recent studies have proven that EA could alleviate substance abuse, inducing anxiety-like behaviors, in people and mice (22, 51).

Of the top 10 cited reference and burst citations (Figure 7C), the article by Bowen et al. has strong burst, sigma, frequency, and half-life that indicates that the article is classical and influential in this field. Bowen et al. proved that mindfulness-based relapse prevention is an effective and safe intervention to reduce relapse and provided preliminarily evidence for MM as well (52). Zgierska et al. first conducted a systematic review on MM, suggesting that MM interventions are safe and effective, although it lacked evidence (53). Thus, the top two strongest citation burst references were on the topic of mindfulness. Moreover, half of the top 10 frequencies, two of the top 10 centralities of cited references, and three of the top 14 burst citations were about mindfulness. Mindfulness is usually considered a behavioral therapy in psychology; however, it was previously considered a body–mind therapy or a CAM therapy (54). Mindfulness combined with meditation is a hot cited topic. According to Figure 7B, cited reference has two psychotherapy clusters, mindfulness and cognitive behavior therapy, indicating these therapies may integrate with CAM more closely. The studies by Koob and Volkow from 2010 and 2016 (55, 56) were also highly cited, and they clarified the neurocircuitry of addiction. The two papers with the top 10 frequencies involve multi-substance dependence, such as tobacco or alcohol. From the network of clusters (Figure 7), the Han's acupoint nerve stimulator (HANS) protocol may be widely used in the future, and acupuncture is an independent CAM therapy in this field, which is certainly effective for treating SUDs and has laboratory and clinical evidence (23, 57). The complications and comorbidities of SUDs, HIV (#4), and shock (#12) are widely concerning, as is the burden on global health (#15) and public health policy.

In the last 30 years, there has been significant progress in the use of CAM for treating SUDs. The researchers in this field are interested in CAP or CAM integration with psychotherapy and acupuncture in clinical practice and in understanding the mechanisms of preclinical medicine. The research generally constitutes clinical medicine practice and mechanisms to prove the efficacy of CAM.

### Significance and Limitations

This study sheds light on the dynamic research progress using bibliometric analysis to determine the direction of research and provide accurate information on this field to researchers. This task was accomplished by summarizing the collaboration countries, authors, cited journals, keywords, and references to determine research hotspots and frontiers, which may help understand the complete picture for peer education, training, and scientific research guidance. Today, bibliometric analysis is widely applied in scientific research. However, it is newly emerging in the study of medicine. In this field, early studies used traditional bibliometric methods, not data visualization. Moreover, there is a lack of macroscopical studies on the effectiveness of CAM for treating SUDs in more than one region or with more than one CAM treatment (58, 59). Thus, this study can provide more information for holistic CAM for SUDs.

Regarding limitations, this study searched papers in English collected by the WoSCC, which may have resulted in bias. However, data from the WoSCC are preferable to a bibliotic analysis by CiteSpace and are of high quality. Selected keywords cannot represent all studies in this field, and the papers were limited to a 20-year timeframe, which may have also led to bias. Moreover, the search strategy was based on CAM MeSH terms, including “biofeedback, psychology;” “imagery, psychotherapy;” “psychophysiology;” “spiritual therapies;” and “meditation,” which led partly to psychotherapy belonging to CAP to be included in the study. In addition, psychotherapy is a suggested treatment for each patient with SUDs, which may be combined CAM for SUDs. Thus, the results of psychotherapy may have led to bias. The study could not analyze more elaborate information as it used macroscopical subjects.

## Conclusion

Overall, CAM for SUDs has been evidently increasingly concerning since 2015. Acupuncture and CAP are hotspots in this field. The comorbidity of SUDs has received great attention as well, particularly HIV, depression, and anxiety. The main research comes from developed countries, especially the USA, and developing countries, such as China and India, which are becoming increasingly concerned about CAM for the treatment of SUDs. Researchers prefer evidence-based studies, such as randomized controlled trials and meta-analyses; however, the existing evidence is still insufficient and lacks cross-regional cooperation. Last but not least, integrative treatment, psychotherapy combined with CAM, may be a promising direction for SUDs.

## Data Availability Statement

The raw data supporting the conclusions of this article will be made available by the authors, without undue reservation.

## Ethics Statement

This study was approved by the Ethics Committee of the First Affiliated Hospital of Guangzhou University of Traditional Chinese Medicine (No. ZYYEC-ERK [2020]100), which waived the need for informed consent because of the retrospective study design.

## Author Contributions

JJ: conceptualization, writing–original draft, and formal analysis. CS: methodology, software, and validation. XQ: visualization and investigation. ZJ: conceptualization and supervision. WX: data curation. LL: writing–review and editing. LG: project administration and funding acquisition. All authors contributed to the article and approved the submitted version.

## Funding

This study was supported by Guangzhou Municipal Science and Technology Bureau (Grant No. 202102010505). This research was supported by the Traditional Chinese Medicine Bureau of Guangdong Province (Grant No. 20204005), the Innovative Research Fund of the First Affiliated Hospital of Guangzhou University of Chinese Medicine (Grant No. 2019IIT12), National Natural Science Foundation of China (Grant No. 81574061), and Traditional Chinese Medicine Bureau of Guangdong Province (Grant No. 20211128). These funding organizations had no role in the study design, collection, analysis, interpretation of the data, writing the manuscript, or the decision to submit the paper for publication.

## Conflict of Interest

The authors declare that the research was conducted in the absence of any commercial or financial relationships that could be construed as a potential conflict of interest.

## Publisher's Note

All claims expressed in this article are solely those of the authors and do not necessarily represent those of their affiliated organizations, or those of the publisher, the editors and the reviewers. Any product that may be evaluated in this article, or claim that may be made by its manufacturer, is not guaranteed or endorsed by the publisher.
