# Supplementary material for: Complementary and Alternative Medicine for Substance Use Disorders: A Scientometric Analysis and Visualization of Its Use Between 2001 and 2020
Source: Front Psychiatry. 2021 Nov 5;12:722240. doi: 10.3389/fpsyt.2021.722240 (PMC8604152; doi:10.3389/fpsyt.2021.722240)
Supplement: Supplementary file 1 [file Data_Sheet_1.docx]

**S 1 0#-10# Cluster of keywords about related to CAM for SUDs.**

| **Cluster ID** | **Size** | **Silhouette** | **Mean (Year)** | **Label (LLR)** |
| --- | --- | --- | --- | --- |
| #0(morphine) | 47 | 0.726 | 2006 | relapse, acupuncture, electroacupuncture, psychotherapy, HIV, self-administration, cpp, cue, dopamine |
| #1(complementary and alternative medicine) | 41 | 0.744 | 2005 | trial, electrical nerve stimulation, auricular acupuncture, psychoeducation, mindfulness-based relapse prevention, relapse prevention, cocaine addiction, heroin |
| #2(meta-analysis) | 40 | 0.803 | 2005 | women, youth, culturally sensitive interventions, prevalence, prevention, domestic violence, national epidemiologic survey, psychotherapy, depression, HIV, comorbidity |
| #3(hiv) | 38 | 0.684 | 2006 | trauma, lymphocyte subset, cancer, risk, group, common themes, specific conditions, alcohol use disorder, substance abuse, population, antiretroviral therapy |
| #4(opioids) | 21 | 0.789 | 2007 | pharmacology, pain management, opioid, bulimia nervosa, death, pain, chronic pain, mental health, double blind, anorexia nervosa, posttraumatic stress disorder |
| #5(substance abuse treatment) | 19 | 0.728 | 2005 | stimulant drug use, hplc（high-pressure liquid chromatography, episodic future thinking, effectiveness, opiate , men who have sex with men, standardization, therapeutic process, severe mental illness, construct validity, inpatient treatment, |
| #6(mediation) | 18 | 0.746 | 2006 | alcoholism treatment, psychoactive substance use disorder, methadone patient, randomized clinical trials, sertraline, short-term psychodynamic psychotherapy, obsessive-compulsive syndrome, gambling, cocaine dependence |
| #7(drug) | 13 | 0.859 | 2005 | impact, over the counter, sedation, determinant, xylazine, intoxication, inhalated drug abuse, facial expression, suppression, contemplation, predicting smoking cessation, self-efficacy, sodium oxybate, butyrolactone |
| #8(mediators) | 5 | 0.949 | 2008 | women's treatment, case management, treatment process, moderators, drug abuse, addiction, alcohol, substance abuse, psychotherapy, HIV, depression, treatment, |
| #9(postherpetic neuralgia) | 4 | 0.976 | 2008 | release oral morphine, intrathecal morphine, chronic noncancer pain, primary sensory neuron, neuropathic pain, transdermal fentanyl, nociceptive response, simplex virus type 1, nervous system |
| #10(translational) | 3 | 0.963 | 2007 | genetic, heterogeneity, neuroimaging, animal behavior, circadian rhythm, polygenicity, treatment |

**S 2 Top 10 cited reference related to CAM for SUDs by frequency.**

| Rank | Freq | Burst | Sigma | Representative author | Year | Title | Half-life |
| --- | --- | --- | --- | --- | --- | --- | --- |
| 1 | 182 |  | 1 | American  Psychiatric  Association | 2013 | Diagnostic and Statistical Manual of Mental Disorders | 5 |
| 2 | 61 |  | 1 | Bowen S | 2014 | Relative Efficacy of Mindfulness-Based Relapse Prevention, Standard Relapse Prevention, and Treatment as Usual for Substance Use Disorders A Randomized Clinical Trial | 4 |
| 3 | 37 | 10.78 | 1.09 | Zgierska A | 2009 | Mindfulness Meditation for Substance Use Disorders: A Systematic Review | 7 |
| 4 | 36 |  | 1 | Bogenschutz MP | 2015 | Psilocybin-assisted treatment for alcohol dependence: A proof-of-concept study | 3 |
| 5 | 35 |  | 1 | Witkiewitz K | 2014 | Randomized Trial Comparing Mindfulness-Based Relapse Prevention with Relapse Prevention for Women Offenders at a Residential Addiction Treatment Center | 4 |
| 6 | 35 | 3.43 | 1.13 | Koob GF | 2010 | Neurocircuitry of Addiction | 7 |
| 7 | 35 | 10.19 | 3.14 | Bowen S | 2009 | Mindfulness-Based Relapse Prevention for Substance Use Disorders: A Pilot Efficacy Trial | 6 |
| 8 | 34 |  | 1 | Johnson MW | 2014 | Pilot study of the 5-HT_2A_R agonist psilocybin in the treatment of tobacco addiction | 4 |
| 9 | 31 |  | 1 | Whiteford HA | 2013 | Global burden of disease attributable to mental and substance use disorders: findings from the Global Burden of Disease Study 2010 | 5 |
| 10 | 30 |  | 1 | Chiesa A | 2014 | Are Mindfulness-Based Interventions Effective for Substance Use Disorders? A Systematic Review of the Evidence | 3 |

**S 3 Top 10 cited reference related to CAM for SUDs by Centrality.**

| Rank | Centrality | Burst | Sigma | Representative author | Year | Title | Half-life |
| --- | --- | --- | --- | --- | --- | --- | --- |
| 1 | 0.15 |  | 1 | Gifford EV | 2006 | Acceptance and relationship context: a model of substance use disorder treatment outcome | 6 |
| 2 | 0.13 |  | 1 | Witkiewitz K | 2010 | Depression, craving, and substance use following a randomized trial of mindfulness-based relapse prevention. | 7 |
| 3 | 0.13 |  | 1 | Morgenstern J | 2000 | Cognitive–behavioral treatment for alcohol dependence: a review of evidence for its hypothesized mechanisms of action | 8 |
| 4 | 0.13 |  | 1 | Morgenstern J | 2007 | Rethinking the paradigms that inform behavioral treatment research for substance use disorders | 1 |
| 5 | 0.12 | 10.19 | 3.14 | Bowen S | 2009 | Relative Efficacy of Mindfulness-Based Relapse Prevention, Standard Relapse Prevention, and Treatment as Usual for Substance Use Disorders  A Randomized Clinical Trial | 6 |
| 6 | 0.12 |  | 1 | Azbel L | 2013 | Burden of Infectious Diseases, Substance Use Disorders, and Mental Illness among Ukrainian Prisoners Transitioning to the Community | 3 |
| 7 | 0.12 |  | 1 | Carroll KM | 2004 | Efficacy of Disulfiram and Cognitive Behavior Therapy in Cocaine-Dependent Outpatients  A Randomized Placebo-Controlled Trial | 3 |
| 8 | 0.11 |  | 1 | Burke BL | 2003 | The efficacy of motivational interviewing: A meta-analysis of controlled clinical trials. | 2 |
| 9 | 0.1 |  | 1 | Rehm J | 2009 | Global burden of disease and injury and economic cost attributable to alcohol use and alcohol-use disorders | 8 |
| 10 | 0.1 |  | 1 | Allen JP | 1997 | Matching alcoholism treatments to client heterogeneity: Project MATCH posttreatment drinking outcomes | 7 |
